# An integrative and multi-indicator approach for wildlife health applied to an endangered caribou herd

**DOI:** 10.1038/s41598-023-41689-y

**Published:** 2023-10-02

**Authors:** Xavier Fernandez Aguilar, Lisa-Marie Leclerc, Kugluktuk Angoniatit Association, Ekaluktutiak Hunters & Trappers Organization, Olokhaktomiut Hunters & Trappers Committee, Fabien Mavrot, Amélie Roberto-Charron, Matilde Tomaselli, Gabriela Mastromonaco, Anne Gunn, Mathieu Pruvot, Jamie L. Rothenburger, Niroshan Thanthrige-Don, Elham Zeini Jahromi, Susan Kutz

**Affiliations:** 1 Faculty of Veterinary Medicine, University of Calgary, Calgary, AB T2N 4Z6 Canada; 2 Department of Environment, Government of Nunavut, P.O. Box 377, Kugluktuk, NU X0B 0E0 Canada; 3 Kugluktuk Angoniatit Association, 13 Klengenberg St., Kugluktuk, NU X0B 0E0 Canada; 4 Ekaluktutiak Hunters & Trappers Organization, 4 Mitik St., Cambridge Bay, NU X0B 0C0 Canada; 5 Olokhaktomiut Hunters & Trappers Committee, P.O. Box 161, Ulukhaktok, NT X0E 0S0 Canada; 6 Polar Knowledge Canada, Canadian High Arctic Research Station, 1 Uvajuq Road, PO Box 2150, Cambridge Bay, NU X0B 0C0 Canada; 7 Reproductive Science, Toronto Zoo, Toronto, ON M1B 5K7 Canada; 8 CircumArctic Rangifer Monitoring and Assessment (CARMA) Network, 368 Roland Rad, Salt Spring Island, BC V8K 1V1 Canada; 9 Canadian Wildlife Health Cooperative (Alberta Region), Alberta, Canada; 10 Canadian Food Inspection Agency, Ottawa Laboratory Fallowfield, 3851 Fallowfield Road, Station H, PO Box 11300, Nepean, ON K2H 8P9 Canada; 11 Alberta Centre for Toxicology, University of Calgary, Calgary, AB T2N 1N4 Canada; 12 Present Address: Wildlife Conservation Medicine Research Group (WildCoM), Departament de Medicina i Cirurgia Animals, Universitat Autònoma de Barcelona, 08193 Bellaterra, Spain

**Keywords:** Zoology, Diseases

## Abstract

Assessing wildlife health in remote regions requires a multi-faceted approach, which commonly involves convenient samplings and the need of identifying and targeting relevant and informative indicators. We applied a novel wildlife health framework and critically assessed the value of different indicators for understanding the health status and trends of an endangered tundra caribou population. Samples and data from the Dolphin and Union caribou herd were obtained between 2015 and 2021, from community-based surveillance programs and from captured animals. We documented and categorized indicators into health determinants (infectious diseases and trace elements), processes (cortisol, pathology), and health outcomes (pregnancy and body condition). During a recent period of steep population decline, our results indicated a relatively good body condition and pregnancy rates, and decreasing levels of stress, along with a low adult cow survival. We detected multiple factors as potential contributors to the reduced survival, including *Brucella* suis biovar 4, *Erysipelothrix rhusiopathiae* and lower hair trace minerals. These results remark the need of targeted studies to improve detection and investigations on caribou mortalities. We also identified differences in health indicators between captured and hunter sampled caribou, highlighting the importance of accounting for sampling biases. This integrative approach that drew on multiple data sources has provided unprecedented knowledge on the health in this herd and highlights the value of documenting individual animal health to understand causes of wildlife declines.

## Introduction

Highly seasonal and high latitude regions such as the Arctic are especially vulnerable to the effects of accelerating climate warming, such as changes in plant composition and phenology^1,2^, shifting wildlife and parasite distributions and communities^3,4^, and altered host-parasite interactions^5^. These changes can exceed the resilience of wild animal populations, resulting in increased morbidity and mortality^6–8^. The remoteness of the Arctic makes health surveillance and investigations especially challenging. Multi-faceted approaches are required to track wildlife health, identify emerging threats, and implement wildlife conservation measures^9^.

The majority of migratory tundra caribou (*Rangifer tarandus*) herds in North America have declined since the early 2000s, with only a few herds currently stable or increasing^10^. About 2.6 to 4.7 million caribou have disappeared from the Arctic in the last two decades, with an average herd population decline of 71% ± 5.9SE from their peak (11 herds with sufficient data), and some of the herds have plummeted by more than 90%^10,11^. Although both traditional and scientific knowledge documented historical fluctuations of tundra caribou^12,13^, several herds are now below any historically documented population levels and show few signs of recovery^10^.

The magnitude of the migratory tundra declines is in stark contrast with our poor understanding of the drivers of the declines. While climate change may be an important driver of caribou population trends^14^, the specific mechanisms causing the declines are poorly understood. This is especially true for the role of nutrition, disease and parasites, and how these health determinants interact with each other^15^. Demographic indicators of caribou populations are regularly monitored, yet these alone are insufficient to understand the underlying drivers of declines. Deeper and comprehensive health investigations are necessary to improve our understanding on how climate change is altering the Arctic systems, identify health and demographic drivers, anticipate threats and, if possible, mitigate them through evidence-based management decisions.

Recent partnerships with Indigenous communities have progressed to establish health surveillance systems through sustained and standardized community-based wildlife health monitoring programs. These programs greatly expand on the sporadic sampling derived from caribou captures and collaring operations^16,17^. In fact, community-based surveillance and local knowledge can detect shifts in health and drivers that may not be detected by other classical monitoring methods that are more restricted by budget or the frequency and seasonality of observations^7,9^. Peacock et al. proposed an integrative framework that incorporates different health metrics informed by local and scientific knowledge to quantitatively assess changes in Arctic wildlife health. This framework is based on benchmarks that are yet to be defined for caribou and baseline data are much needed^17^.

The complexity of multiple factors influencing health is conceptually represented by the Determinants of Health model, which was conceived for human health and has been adapted to wildlife^18^. These conceptual models acknowledge the accumulative effects of a wide range of determinants, yet do not clearly differentiate between causes and consequences. They also do not include previously discussed concepts on wildlife health such as the differentiation between determinants and outcomes^19^. With the aim to operationalize health assessments in wildlife, we use a novel framework that classifies health indices according to the type of information they provide.

The goal of our research was to build on existing wildlife health frameworks to evaluate the significance of various individual animal health indices for understanding the health of an endangered tundra caribou herd, the Dolphin and Union herd (*Rangifer tarandus groenlandicus* × *R. t. pearyi*). This caribou herd has experienced recent steep population declines and since 2015, monitoring efforts including capture and collaring events and community-based surveillance have been intensified, generating an unprecedented amount of health information on this herd. We compiled this information and applied a structured approach to classify and analyze different health indices into health determinants, health processes, and health outcomes. With this study, we aim to share experiences on this approach, identify mechanisms and drivers of the recent caribou herd decline, identify relevant health indicators and further surveillance needs on caribou health, and critically assess the combination of community-based sampling and sampling from captured animals for health investigations.

## Results

### Dolphin and Union caribou sampling

**Figure 1:**
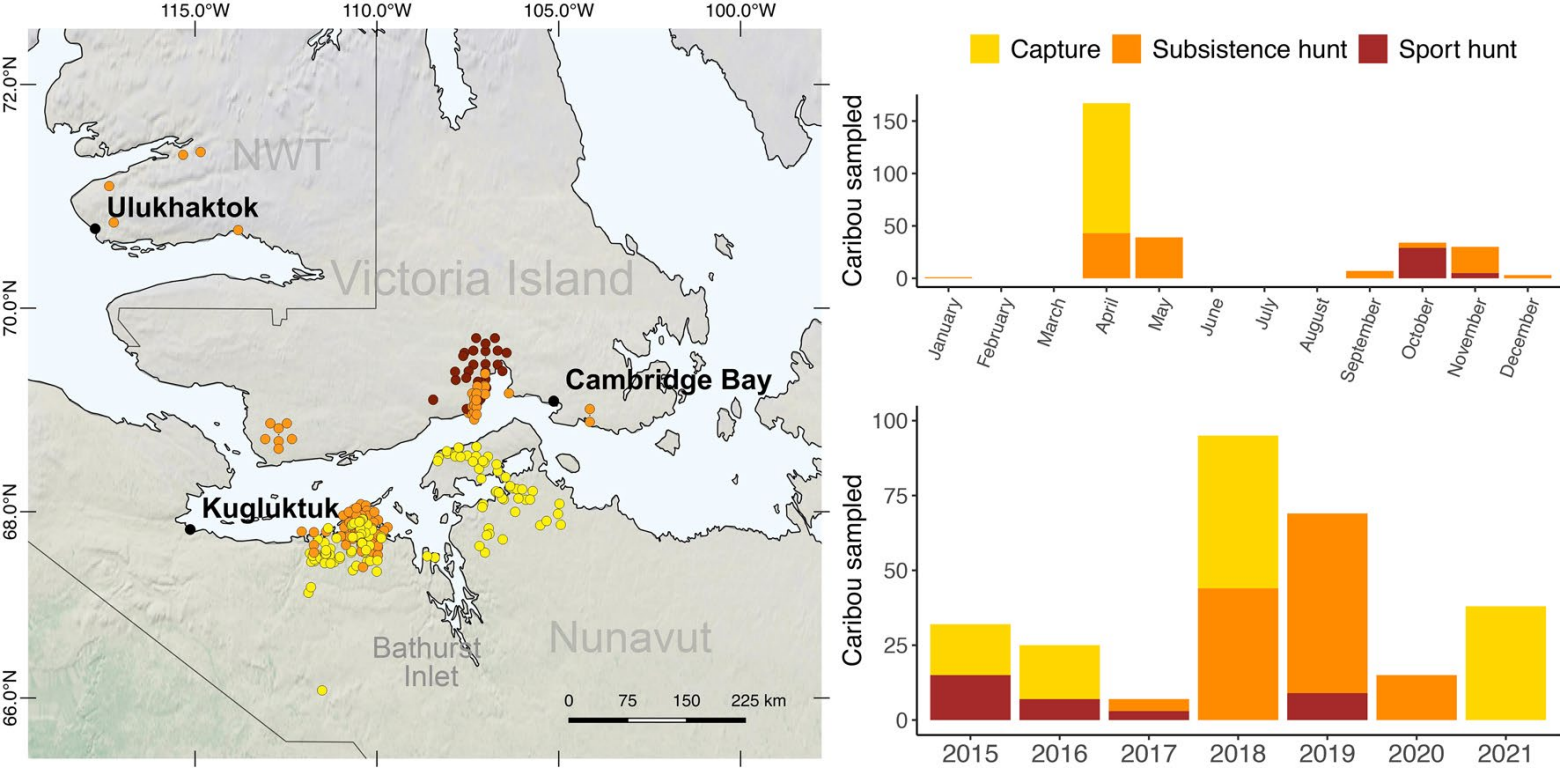
Location and number of samples collected for health surveillance of the Dolphin and Union caribou herd. Left: locations of the Dolphin and Union (DU) caribou sampled since 2015 in Nunavut and Northwest Territories (Canada), and the three communities that are involved in harvester-based sampling. Right: Number of DU caribou sampled over the years by sampling source. The map was produced using QGIS (QGIS Development Team 2016, Las Palmas 2.18.15; QGIS Geographic Information System; Open-Source Geospatial Foundation Project).

A total of 298 caribou were sampled between 2015 and 2021 (Table S1, Fig. 1). Seventeen animals were eliminated from the dataset after genetic analyses revealed they had barren-ground genetics (> 50% genetic profile, Supplementary Appendix S6). Sample submissions varied by year and in part due to availability of specific funding (subsistence hunts from 2015 to 2017) and disruption of sampling during the COVID-19 pandemic (2020). Sport hunts on the DU caribou ceased in 2019 in response to low population estimates, and samples from captured caribou (adult females) were only available for the years when the Government of Nunavut were deploying GPS collars.

Most of the samples were collected in early spring (73% from mid-April to mid-May), and less commonly, in the fall (25% from mid-September to mid-November). These two periods are when the DU herd is closer to communities and when most of the harvests and captures (spring only) happen. This seasonal sampling helped to standardize data within and across years, but limits insight into the calving and summer period.

Historical health information on the DU herd included data from 162 adult caribou cows sampled in early spring between 1987 and 1991 (n = 91) and 2001–2003 (n = 71) (see “Materials and methods” section for further details).

### Results on health outcomes

**Figure 2:**
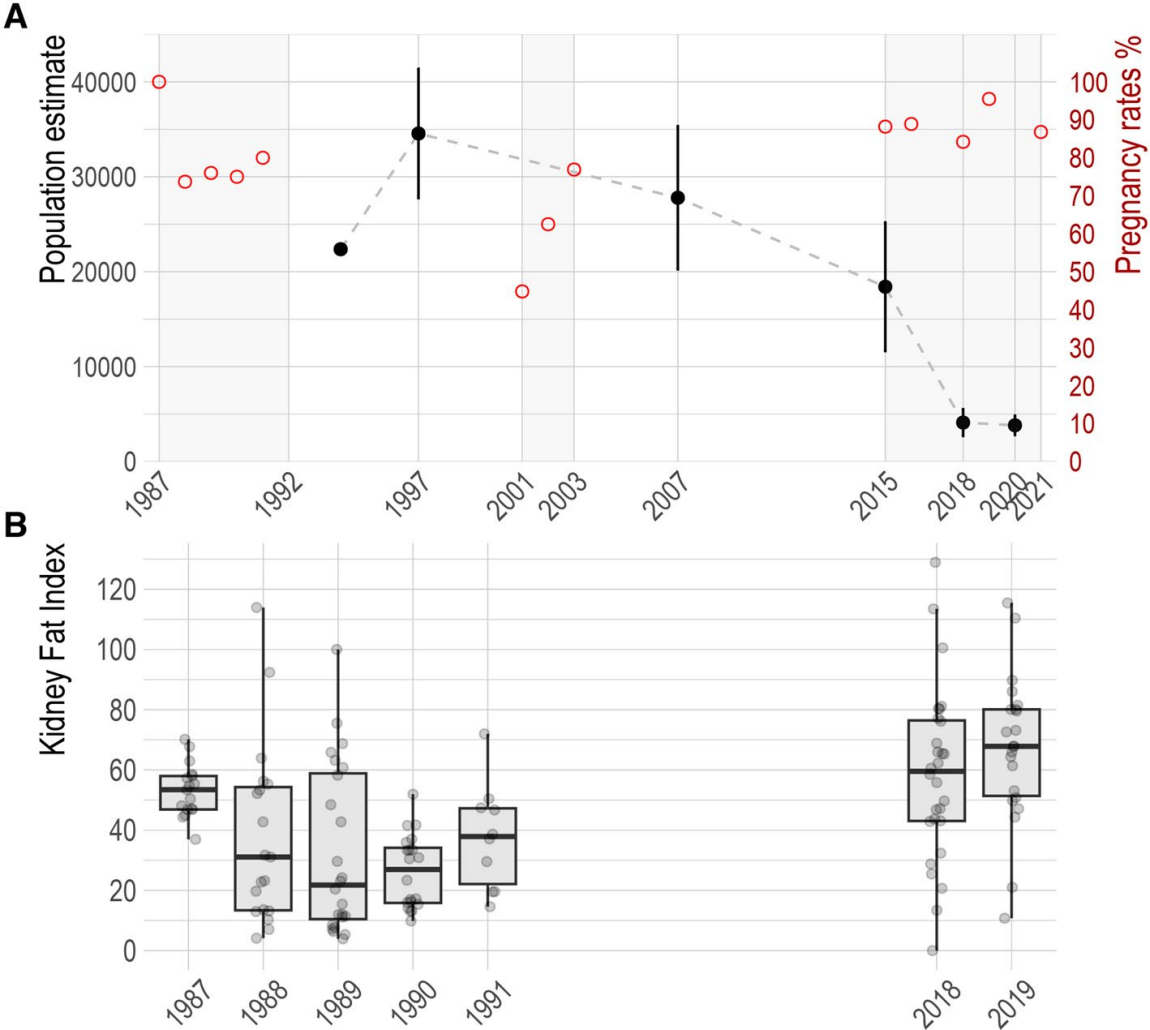
Long-term data on health outcomes in the Dolphin and Union caribou herd. (**A**) Black dots and dashed line indicate population trends of the Dolphin and Union caribou herd (left axis), according to the Government of Nunavut population aerial surveys^20,21,22^. Range black lines on the dots are the standard error of the estimates. Red dots indicate the pregnancy rate estimated for that year (right axis), in three different time periods highlighted with a grey background. These pregnancy rates include estimates from adult females in early spring obtained in this study (hunted caribou: 2018 and 2019 and captured caribou: 2015, 2016, 2018, 2021), previous government surveys (hunted caribou 1987–1991) and research works (hunted caribou 2001–2003). (**B**) Distribution of Kidney Fat Index by year, when available, from the same dataset as the upper chart. Data points are overlaid on the boxplots to show sample size by year.

The average pregnancy rate in adult caribou sampled in early spring for the period 2015–2021 was 86.8% (95% CI 80.9–91.1). In 2018, pregnancy rates were significantly lower in hunted 67.9% (95% CI 49.3–82.1) vs captured 93.7% (95% CI 83.2–97.9) caribou (n = 76, p = 0.007 Fisher’s exact test). Among captured caribou in 2018, no differences were detected between animals captured West of Bathurst Inlet (30 pregnant out of 32 adult cows sampled) and East of Bathurst Inlet (30 pregnant out of 32 adult cows sampled) (Fig. 1). For the period 2015–2019, the average pregnancy rate of hunted adult females in early spring (78.7%; 95% CI 61.8–83.1) was similar to that documented for the period 1987–1991 (76.2%; 95% CI 67.3–82.7), but significantly higher than for the period 2001–2003 (57%; 95% CI 46.5–67.5, *X*^2^ (df = 2, n = 212) = 8.8, p = 0.01) (Fig. 2).

**Figure 3:**
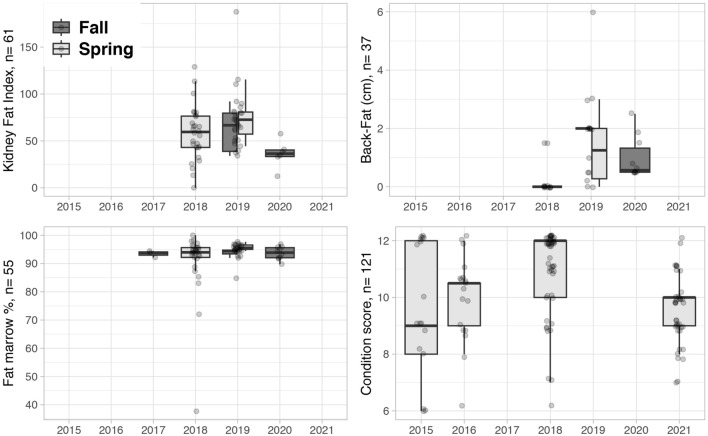
Distribution and number of observations of four different body condition metrics in adult Dolphin and Union caribou females. Data points are overlaid on the boxplots to show sample size by year. Years with similar values appears as horizontal black lines (back-fat spring 2018 and fall 2019, fat marrow fall 2017). Kidney Fat index, Back-Fat and Fat marrow % are metrics from hunted caribou and condition score from captured caribou. The hunter qualitative assessment of body condition is not represented in this figure because it had low number of observations.

The qualitative assessment of body condition by hunters and back-fat measurement were not consistently recorded on datasheets. Therefore, body condition data were mostly represented by the condition score (caribou captures) and by Kidney Fat Index and Fat marrow from females harvested at early spring (April and May) (Fig. 3). Differences between years were only assessed for the condition score in captured caribou because it was the metric with the most years of data for adult females sampled during the same season. The distribution of the condition score differed significantly by years in a Kruskal Wallis test H (df = 3, n = 121) = 23.2, p < 0.01, with a higher median value in 2018 (12.0) than previous (2015: 9.0; 2016: 10.5) and later years (2021: 10) (Fig. 3).

Among adult females sampled in early spring, KFI median values in 2018–2019 (KFI: 64.8, n = 50) were statistically higher than in 1987–1991 (KFI: 34.7, n = 90), H (df = 1, n = 140) = 28.06, p < 0.001 (Fig. 2).

### Results on host response metrics

**Figure 4:**
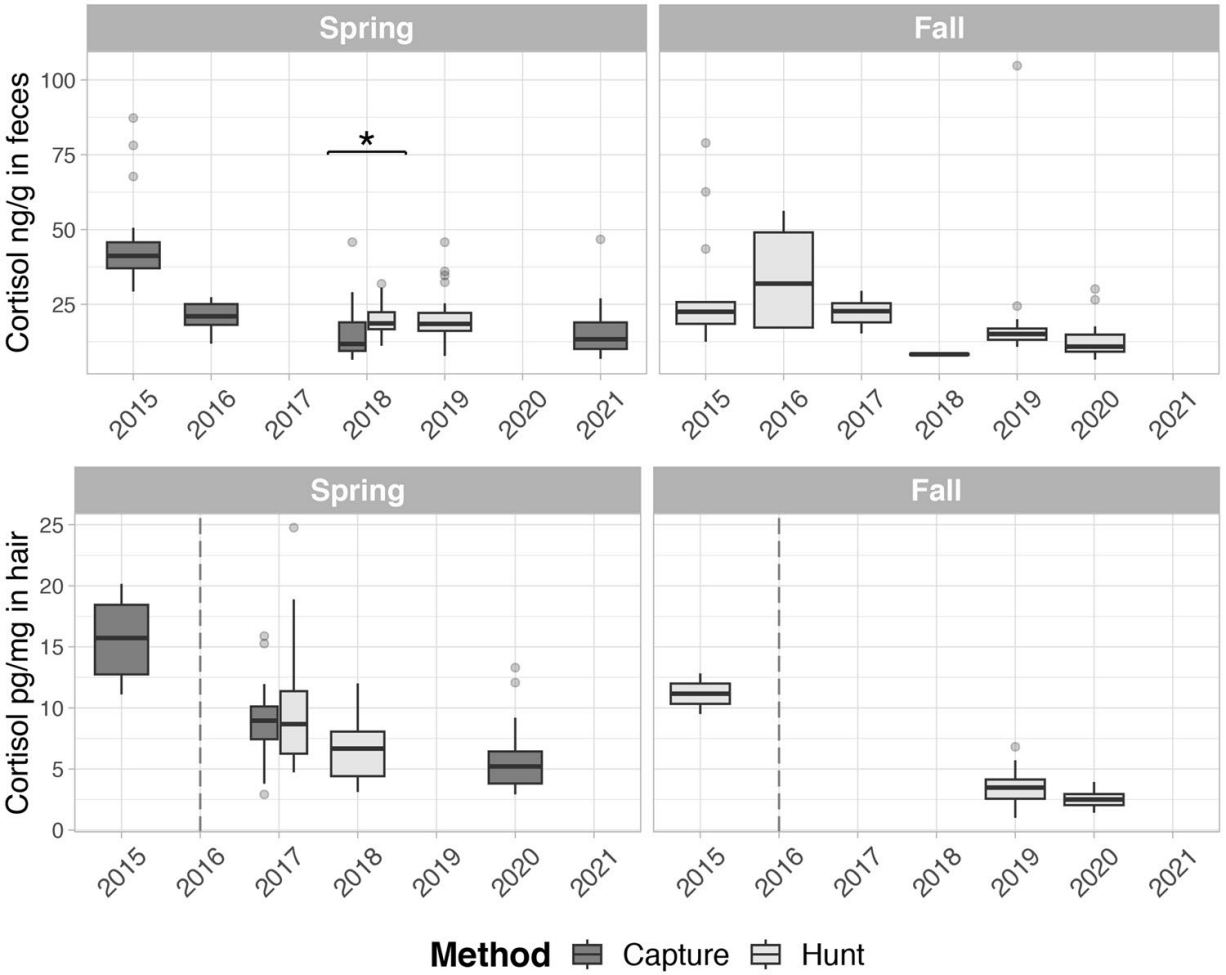
Trends and distributions of cortisol concentrations from adult Dolphin and Union caribou in feces and hair. Results of cortisol concentrations in feces (upper panel) and in hair from the neck (lower panel), showed by season and by the sampling method used to obtain the samples. Results of hair cortisol are plotted by the year in which the hair was grown. Cortisol data in feces from the fall 2018 appears as a dark horizontal line because it is only composed by two similar values (8.6 and 8.0 ng/g). The dashed lined in the lower panel graphs separates results that were analyzed in different laboratories and methods (2015 vs 2017–2020). Significant differences between the method of sampling are indicated with an asterisk.

Data were assessed separately when hair was analyzed in different laboratories and methods (2015 vs 2017–2020). In adult caribou, the median hair cortisol value in 2015 was 14.7 pg/mg (min: 9.5–max: 20.2) and for the period 2017–2020 it was 6.7 pg/mg (min: 1.0–max: 24.8). Internal research with a blinded interlaboratory comparison indicated that a 0.64 average conversion factor could be applied to compare results from the two methods/laboratories^23^, which would still lead to a higher hair cortisol, 9.4 pg/mg in 2015, vs 8.8 pg/mg in 2017 and 4.4 pg/mg in 2020 (Fig. 4).

We found no significant differences in hair cortisol between harvested vs captured, or sex, using a subset of data from adult caribou sampled in early spring 2018. It was not possible to assess the differences of hair cortisol between sampling sources (harvest vs capture) in other years because it was confounded with season (Fig. 4). Within data from 2017 to 2020, hair cortisol in early spring was significantly lower in later years for both captured (2017 vs 2020: n = 89, Z =  − 5.38, p < 0.01), and hunted (2017 vs 2018: n = 61, Z =  − 3.03, p < 0.01) adult caribou (Fig. 4).

The overall median value for fecal cortisol in adult caribou was 17.73 ng per g of wet feces (min: 6.2–max: 104.7). Fecal cortisol had no significant differences between males and females, but was higher in hunted (18.62 ng/g wet feces) than captured caribou (median 11.76 ng/g wet feces) in a subset of data with adult caribou from early spring 2018 (n = 80, Z =  − 3.74, p < 0.01). Among adult caribou captured in early spring, fecal cortisol varied significantly between years, H (df = 3, n = 135) = 49.6, p < 0.01. Wilcoxon pairwise comparisons with a Bonferroni correction indicated that these yearly differences occurred between all years except for 2018 and 2021. Median fecal cortisol decreased over the years (2015: 41.2 ng/g of wet feces; 2016: 21.0 ng/g; 2018: 11.8 ng/g; 2021:13.34) (Fig. 4).

#### Results on health determinants

We detected exposure of DU caribou to all the pathogens tested (Table 1). Sample prevalence of antibody detection was different depending on the sampling method only for *Brucella*, which was higher in hunted caribou (34.3%; 95% CI 20.8–50.8) than captured caribou (9.8%; 95% CI 4.3–21.0) (n = 86, Fisher’s exact test p = 0.01), assessed in a subset of data from adult caribou sampled in early spring 2018. Sample prevalence for pathogen exposure varied significantly by year using Fisher’s exact tests, for all pathogens except for *Brucella* and Pestivirus (Table 1).

**Table 1:** Sample prevalence and confidence intervals for parasites and pathogens in adult Dolphin and Union caribou.

	2015^a^	2016^a^	2017	2018	2019	2020	2021	Total
Antibody detection—sample prevalence % (CI 95%)								
Pestivirus	29.2 (14.9–49.2)	21.7 (9.7–41.9)	0.0 (0–39.0)	23.6 (16.0–33.4)	13.9 (6.6–27.3)	15.4 (4.3–42.2)	8.3 (2.9–21.8)	18.8 (14.3–24.3)
α-Herpesvirus*	85.7 (68.5–94.3)	100 (81.6–100)	NA	83.1 (74.0–89.5)	83.7 (70.0–91.9)	15.4 (4.3–42.2)	75.0 (58.9–86.2)	79.6 (73.9–84.3)
*Brucella* spp.	10.7 (3.7–27.2)	13.0 (4.5–32.1)	0 (0–39.0)	19.1 (12.3–28.5)	20.9 (11.4–35.2)	7.7 (0.4–33.3)	13.5 (5.9–28.0)	16.3 (12.2–21.5)
Erysipelothrix rhusiopathiae*	21.4 (10.2–39.5)	36.36 (19.7–57.0)	33.3 (9.7–70.0)	19.1 (12.3–28.5)	6.9 (2.4–18.6)	0 (0–22.8)	16.2 (7.6–31.1)	17.6 (13.3–23.0)
Neospora caninum*	8.3 (2.3–25.8)	33.3 (16.3–56.2)	NA	2.2 (0.11–11.6)	0.0 (0–48.9)	NA	0.0 (0.0–9.4)	7.0 (3.7–12.8)
Parasite detection—sample prevalence % (CI 95%)								
*Besnoitia tarandi* (skin)	NA	33.3 (1.7–79.2)	40 (11.8–76.9)	39.4 (24.7–56.3)	43.2 (28.7–59.1)	NA	NA	41.0 (30.8–52.1)
Protostrongylid Larvae/gram (feces)^#^	5.9 (0.3–27.0)	22.2 (9.0–45.2)	NA	7.5 (3.5–15.4)	11.54 (4.0–29.0)	NA	9.1 (3.1–23.6)	9.8 (6.2–15.1)

These results include samples collected both by hunters and during caribou captures.

*NA* no samples/data available.

^a^Part of the antibody detection results were previously published in Ref. ^24^, but data has been updated to include hunted caribou and exclude animals without DU caribou genetics.

*Differences between years were statistically significant using Fisher’s exact tests.

^#^Adult animals sampled in early spring (April–May).

We found significant differences of sample prevalence between adult males and females only for Herpesvirus exposure (males: 93.0% and females: 76.5%, n = 223, Fisher’s exact test p = 0.018). Because the proportion of males and females sampled per year varied, we further confirm differences in herpesvirus exposure by sex with a subset of data that only included hunted caribou and excluded data from 2020 (males: 97.3%, females: 71.9%, n = 94, Fisher’s exact test p < 0.01), which was the only year in which the sample prevalence on herpesvirus was statistically different from other years (Table 1).

*Besnoitia* cysts were detected in the metatarsus skin in 40.0% (95% CI 31.1–49.6) of the caribou analyzed (n = 105), with a median cyst density of 0.24 cysts/mm^2^ (min.: 0.039–max.: 1.059). *Besnoitia* cyst detection in adult caribou did not vary significantly by sex or year (Table 1). Low intensities of protostrongylid larvae per gram of feces (min.: 0.20, median: 3.75, max.: 148.32) of the parasites *P. andersoni* and/or *V. eleguneniensis* were found in 9.9% (95% CI 6.0–16.0) of the fecal samples analyzed (n = 141). Protostrongylid fecal larvae detection in adult caribou in spring did not significantly vary by the method of sampling, sex or year (n = 174, Table 1), and parasite counts were aggregated in few individuals following a negative binomial distribution.

In the hair elements analysis, several elements had more than 40% (Na, Mg and K) or 90% (Cr and Co) of their values below the limit of quantification (LOQ) of the technique and no further assessments were possible. Among the heavy metals that are not trace minerals, As was not detected in any sample, Cd was detected in two samples above the LOQ (min: 0.26 ppm; max: 0.33 ppm) and Pb levels were above the LOQ in 57% of the samples (min: 0.02; median: 0.05; max: 50.8). There were no significant differences between sex of the animals in any of the elements analyzed using subsets of adult caribou sampled in both spring 2018 and data from all years, but there were significant differences in Fe between captured (median: 8.8) and hunted (median: 10.6) caribou, in a subset of data that included adult caribou sampled in spring 2018 (n = 85, Z =  − 2.78, p < 0.01). Apart from selenium, there were significant differences in all hair trace minerals among years using Kruskal–Wallis tests in the subset of data of adult caribou sampled in spring (n = 129, Table 2).

**Table 2:** Concentration of trace minerals in parts per million (ppm) measured in hair from the neck of adult Dolphin and Union Caribou sampled in early spring.

Element	2017 (n = 85)			2018 (n = 26)			2020 (n = 18)			Total (n = 129)		
	Min.	Median	Max.	Min.	Median	Max.	Min.	Median	Max.	Min.	Median	Max.
Ca*	202.0	373.7	930.9	178.7	335.3	1004.2	295.1	362.8	554.0	178.7	362.0	1004.2
Mn*	0.154	0.464	1.794	0.319	0.550	1.369	0.226	0.302	0.583	0.154	0.459	1.794
Fe*	4.608	9.070	254.0	6.877	11.61	108.26	7.590	9.510	21.70	5.563	9.552	254.0
Cu*	4.414	6.600	13.68	3.904	5.899	7.574	5.627	6.452	7.602	3.904	6.486	13.68
Zn*	63.31	85.66	153.4	60.91	82.45	109.18	63.75	78.24	89.43	60.91	83.34	153.42
Se	0.102	0.231	0.488	0.167	0.216	0.348	0.169	0.208	0.378	0.102	0.223	0.488
Mo*	0.031	0.064	0.704	0.036	0.056	0.122	0.0396	0.077	0.108	0.032	0.066	0.704

Element concentrations in hair are shown in the corresponding hair-growing year and not in the year in which the samples were collected.

*Differences between years were statistically significant (p < 0.05) using Kruskal–Wallis tests.

#### Abnormalities diagnosed during the sampling program

Between 2015 and 2021, eight cases of abnormal findings were submitted by hunters for pathological examination and were mostly diagnosed as brucellosis (Table 3). Among captured caribou, white-to-translucent spherical nodules in the eyes, consistent with *B. tarandi* cysts in the ocular sclera, were recorded in 8 out of 107 animals (not examined in 2015), and a one hindlimb hoof was overgrown in a cow captured in 2021 (negative to *Brucella* antibodies).

**Table 3:** Cases of abnormalities in Dolphin and Union caribou submitted through the hunter-based sampling program between 2015 and 2021.

Animal ID	Date	Sampling source	Abnormality detected	Sample submitted/gross findings	Etiologic diagnosis*
DU-104	2018-05-12	Hunter-based sampling	Swollen joints in both front legs	Two forelimbs/bursitis and hygromas in both carpometacarpal joints	Brucellosis (isolation of *Brucella suis* biovar 4)
DU-141	2018-05-08	Hunter-based sampling	Swollen joint in one front leg	One forelimb/bursitis in metatarsophalangeal joints	Brucellosis (isolation of *Brucella suis* biovar 4)
DU-201	2018-05-03	Hunter-based sampling	Swollen joints in both front legs	One forelimb/bursitis and hygromas in both carpometacarpal joints	Brucellosis (isolation of *Brucella suis* biovar 4)
DU-233	2019-05-12	Hunter-based sampling	Swollen testicles and joint in one front leg	Testicles and one forelimb/orchitis and bursitis in the carpometacarpal joint	Brucellosis (isolation of *Brucella suis* biovar 4)
DU-236	2019-05-13	Hunter-based sampling	Hard, small lump in each front leg	One forelimb/bone overgrowth around carpometacarpal joint, consistent with chronic inflammation	Brucellosis (isolation of *Brucella suis* biovar 4)
DU-236	2019-05-13	Hunter-based sampling	Liver nodules	Piece of liver/parasitic cysts	Hydatid cysts compatible with *Echinococcus canadensis*
DU-284	2019-05-06	Hunter-based sampling	Lumps in the lung	Piece of lung/parasitic cysts	Hydatid cysts compatible with *Echinococcus canadensis*
CBDU004	2020-11-01	Hunter-based sampling	Liver nodules at section	Piece of liver/granulomatous lesions	Brucellosis (isolation of *Brucella suis* biovar 4)

These cases were submitted to the Diagnostic Service Unit, Faculty of Veterinary Medicine at the University of Calgary for diagnostic investigations by veterinary pathologists.

*Diagnosis was achieved using a combination of gross lesions, histopathology and ancillary testing. *Brucella suis* was confirmed by the Brucellosis Centre of Expertise from the Canadian Food Inspection Agency (CFIA) in Ottawa, Canada.

## Discussion

Wildlife health in its broad and modern definition, involves the capacity of animals to cope with stressors and maintain basic physiological functions, such as reproduction and survival^19,25^. Assessing wildlife health in remote regions requires a multi-faceted approach, often including the use of diverse convenience sampling sources, and identifying and targeting relevant and informative indicators. We investigated multiple health indices in the endangered Dolphin and Union caribou herd over a 7-year period using samples from a collaborative health surveillance program that includes data and samples obtained by caribou harvesters and from capture events for monitoring purposes.

We classified our metrics on health into categories of outcomes, processes (host responses) and determinants^26^. This provides a practical framework to assess health in caribou when using multiple indicators, and complements previous frameworks that have been proposed^17,18^. Health outcomes such as reproduction, survival and body condition, are important indicators of caribou fitness which ultimately determine vital rates and population trends. As such, health outcomes are among the most important and immediate information to understand the trajectory of wild populations and relevant for guiding management decisions. However, to understand mechanisms and the fundamental drivers of caribou declines, and to anticipate changes in population trajectory, it is important to examine health determinants and associated host responses in relation to these outcomes. This framework is broadly applicable, but is particularly valuable in remote wildlife populations from which population estimates and access to detailed examinations and investigations are limited.

Our results suggest that body condition and pregnancy rates were unlikely the cause of the accelerated declines, rather, the decline was more likely linked to health determinants that affected adult survival, and perhaps calf survival^20^. The overall pregnancy rate in DU caribou between 2015 and 2021, which includes the time of the steepest documented decline in this herd (between 2015 and 2018), was similar to other caribou herds from North America that were increasing or relatively stable^27^. Pregnancy rates and body condition from harvested DU caribou in these recent years, were similar to those documented in the late 80s, when the herd was increasing, and better than those in the early 2000s, when it had begun to decline (Fig. 2, Ref. ^13^). In contrast, data from collared adult cows indicated a lower survival rate in the most recent years, 0.70 in 2015 and 0.62 in 2018^20,21^, when compared to the rate 0.76 obtained for the period 1999–2004^28^. Hunting by communities contributed to the DU caribou mortality, but when this cause was removed in survival analysis, the survival rate in 2015 remained low (0.72) and still consistent with a declining population^20^. Local knowledge of this herd indicates that the herd started to decline steadily around 2000s when skinnier animals and higher presence of diseased animals started to be noted^7,13^. This is consistent with the pregnancy results recorded in early 2000s. However, the better pregnancy rates and body condition documented in the recent years suggest that different processes may have driven the steep decline between 2015 and 2018. Demographic studies on barren-ground herds found that herd size was most sensitive to adult survival^29,30^, and similar to our results, adult female survival was considered among the most important vital rate to explain the rapid decline of the Bathurst herd between 2006 and 2009^30^.

Cortisol concentrations in DU caribou hair and feces had a peak at the beginning of the study period and decreased over the years. Hair cortisol most likely reflected cumulative stressors during the main hair growth period (summer), or perhaps throughout the year^31^, yet a significant decrease was documented. This pattern followed by both faecal and hair cortisol is coincident with the most accelerated declines of the DU caribou herd and the subsequent stabilization in the last population estimates, between 2018 and 2020, at low numbers. Cortisol is a hormone that reflects the hypothalamic–pituitary–adrenal axis activity^32^, and is a non-specific host response to stressors. It has been associated with different environmental stressors and disturbances in ungulates, including harvest and management practices, population density or demographics^33^, but not with internal macroparasites^32^. In DU caribou, higher allostatic loads seem to have been concurrent with the accelerated decline of the herd. Although this trend may be linked to the stressors that caused the fast herd decline, it could also be reflective of density-dependent mechanisms.

We detected exposure to parasites (micro- and macro-) that can potentially affect caribou health either through: direct reproductive loss (*Brucella*, pestivirus, *N. caninum*, possibly α-herpesvirus); indirect effects through energetic costs (macroparasites), and; causing severe disease or death (mostly microparasites). Our results from health outcomes suggest that determinants that affect survival may have been more important in the most recent period. Therefore, we focus our assessments on microparasites that are more likely to cause severe health effects or death in caribou. However, it should be noted that antibody detection of pathogens that cause significant mortality after exposure may lead to an underestimation of its circulation, as positive animals would only be detected if they had recovered or survived from the infection.

The DU caribou had higher antibody prevalence against of *Brucella* and *α-herpesvirus* compared to recent estimates in other tundra caribou herds^24,34^. The effects of these pathogens in *Rangifer* are described in Table 4. *Brucella suis* biovar 4 was the main infectious agent isolated from abnormalities submitted by hunters during the study period (Table 3). Although sporadic, these lesions are consistently reported in caribou herds affected by brucellosis^35^, and we can reasonably assume that at least a fraction of the exposed caribou develop severe lesions that could negatively impact survival.

**Table 4:** Health Indices used in this study for the health assessment of the Dolphin and Union caribou herd.

Health indices and metrics	Type indicator	Main health effects, key characteristics (health determinants)	References
Pregnancy rate	Health outcome	NA	^27^
Body condition—kidney fat index, bone marrow fat %; back-fat; hunter score (not assessed); palpation score	Health outcome	Proxy for fecundity**, NA	^36,37^
Survival*	Health outcome	NA	
Fecal cortisol	Health process	NA	^32,38^
Hair cortisol	Health process	NA	^38^
Lesions	Health process	Abnormal findings by hunters, NA	
Protostrongylid parasite larvae	Health determinant	Proxy for internal parasite numbers, effects are intensity-dependent, energetic costs	^39^
*Besnoitia tarandi* cysts	Health determinant	Dependent on parasitic intensity, may affect survival. Most probably vector-borne and climate linked	^40,41^
Pestivirus antibodies	Health determinant	Reproductive loss, possible sporadic and rare mortality reported in other species. Variability in virulence and highly mutagenic, long-term protective immunity after exposure	^42,43,44^
α-Herpesvirus antibodies	Health determinant	Ocular disease outbreaks and respiratory disease in reindeer but survival effects not demonstrated, possible reproductive loss. Long-term latency and reactivation under other stressors	^45,46^
*Brucella* antibodies	Health determinant	Reproductive loss, severe disease in some caribou but survival effects not demonstrated. Associated with poor reproductive parameters in caribou	^35,47^
*E. rhusiopathiae* antibodies	Health determinant	May affect survival, demonstrated in other Arctic species	^6,48,49^
*N. caninum* antibodies	Health determinant	Reproductive loss, population effects unknown	^39^
Hair trace minerals	Health determinant	Deficiencies or imbalances may affect resilience and physiological functions involved with growth, immunity, reproduction and survival	^50^

The effects on health of determinants are based on available knowledge on caribou or related species, or the known pathobiology of the infectious agents.

*NA* not apply.

*Results are from the Government of Nunavut and are used for the discussion in this study.

**Fecundity as the physiological potential to be pregnant at term.

Harvesters in the 1990’s and early 2000s began reporting clinical signs consistent with brucellosis in the DU caribou herd^7,13^. This corresponded with the initial decline of the herd and the lower pregnancy rates recorded in 2001–2003 (this work). While brucellosis may have contributed to the herd decline in earlier years, the overall good pregnancy rates and body condition suggest that additional health determinants may have been involved during the period of accelerated decline between 2015 and 2018.

*Erysipelothrix rhusiopathiae* has recently been identified as the cause of several mortality events in muskoxen (*Ovibos moschatus*), and has also been isolated from caribou carcasses^48^. Exposure in tundra caribou is common, and summer seroprevalence is positively associated with a range of environmental factors^49^. The seroprevalence in DU caribou was higher in 2016 when the herd suffered its steepest decline and decreased in the subsequent years (Table 1, Fig. 2). Increasing evidence indicates that a virulent strain of *E. rhusiopathiae* is circulating in the Canadian Arctic^6,48^, however, given the opportunistic nature of this pathogen^48^, it cannot be discarded that its transmission and impact may also be driven by other stressors. While we cannot infer conclusive relationships of *E. rhusiopathiae* and health outcomes on the DU caribou herd, this pathogen is a significant cause of mortality and population declines in muskoxen^6^, is shared across multiple species^51^, and could also be involved in caribou mortalities.

The remaining pathogens studied by serology, including pestivirus, *N. caninum* and α-herpesvirus, are less likely to cause significant mortality and are mostly associated with reproductive loss or other type of syndromes (Table 4). However, sporadic mortalities related to increased virulence in circulating pestivirus strains have been documented in other wild ungulates^52,42^. It is noteworthy that the sample prevalence of pestivirus, *E. rhusipathiae*, *N. caninum* and α-herpesvirus decreased along with the herd decline (Table 1). These trends may be caused by the reduction of the herd size and density-dependent mechanisms, decreasing their transmission or exposure due to smaller caribou aggregations. However, the surge in α-herpesvirus seroprevalence followed by a subsequent decline might have been induced by other stress-inducing factors. This is plausible, considering that latency and viral reactivation often accompany heightened stress, a well-known trait of this type of viruses^45^. This hypothesis seems to be supported by the cortisol trends (Fig. 4).

The prevalence and intensity of *Besnoitia tarandi* cysts and protostrongylid larvae were at the low range of that reported in barren-ground caribou herds^40^. There are no reference values for hair trace minerals in caribou and we can only compare our results with those from other *Rangifer* populations. Most element concentrations in the DU caribou were lower than those in a domestic reindeer herd from the University of Calgary (unpublished results, neck location n = 13, medians Ca: 1565.51; Mn: 2.62; Fe: 34.53; Zn: 114.25; Se: 0.52; Mo: 0.24 ppm), and Se was lower than the Bluenose East herd (comparison only with neck location, Se: 0.39 ppm)^31^. In a northern contaminants study of the DU caribou, Cu and Se in kidneys of bulls were lower than those in other barren-ground caribou herds^53^. Together these data suggest that some elements may be lower in the range of DU caribou. Although micronutrient deficiencies are unlikely to directly cause mortality events, they may affect the resilience against other determinants.

Convenience sampling entails a nonrandom selection criterion of the animals. These sampling schemes, as compare to stratified random sampling, may suffer from selection or detection biases because are less likely to incorporate the variability introduced from biology and spatial and temporal scales^54,55^. Selection biases towards healthier animals may happen in both live-captured and harvested animals. Caribou captures were performed to study movements and space use, thus diseased or weak animals were unlikely to be selected within groups. Similarly, Inuit harvesters prefer caribou with good body condition for consumption^56^. We found differences in pregnancy rates, fecal cortisol, and exposure to *Brucella* between captured and harvested animals, suggesting that the selection criteria between these two sampling sources may differ. We can expect that selection criteria in caribou captures performed from a helicopter is less context-dependent than hunter-based sampling, in which the hunter may not have as much opportunity to choose. In fact, clinical brucellosis with skinnier animals and swollen joints were only detected in caribou sampled by hunters (Table 3). Previous studies also found that herd vital rates obtained from collared caribou were better than they should be to explain the herd size trends, as derived from demographic models^30^. All these findings recommend further studies on how much health estimates obtained by convenient sampling schemes deviate from estimates obtained by stratified random samplings.

The higher fecal cortisol in harvested caribou from early spring may be associated with the concentration of hunters on specific areas from the DU migratory route (Fig. 1). These intensive (few weeks) and geographically concentrated hunting efforts, may lead to repeated stress in caribou groups crossing these areas. We also found higher concentration of iron in hair from hunted caribou, but this finding is unexpected and difficult to explain. Other sources of variation in our metrics included the year of sampling for most of the metrics and sex in herpesvirus exposure, which has previously been reported in *Rangifer*^57^. However, samples from other seasons than spring and from males were poorly represented in our dataset and require further assessments.

Data on health outcomes suggest that recent declines of the Dolphin and Union caribou herd were not associated with reduced pregnancy rates or poor body condition in early spring, rather, lower survival may have played a more important role. These results were also consistent when putting them into a broader context of herd population dynamics, by drawing on health data from historical caribou harvests and Indigenous knowledge on the herd. We provide insights into possible determinants and mechanisms that may have affected survival during the steep population decline (i.e. *Brucella suis* biovar 4, *Erysipelothrix rhusiopathiae* and lower trace minerals), yet other determinants such as extreme climatic events may have also contributed to these trends^58^. It is important to note that density-dependent factors, such as predation or factors related to the carrying capacity, are unlikely to explain the recent steep decline in the herd’s trajectory. Recent changes in the herd range, documented by local knowledge^13^, and intermixing with other barren-ground (see genetic analyses, Supplementary Appendix S6), may have exposed this herd to changes or additional determinants that could have played a role in the observed trends. Complimentary approaches and targeted studies are needed to improve detection and investigations on caribou mortalities, for example, by investigating causes of death in collared animals.

The synergistic approach of harvester-based sampling and captures provided an increased sample size and different types of complementary information over different spatial and temporal scales, which is critical given the high individual, seasonal and yearly variability of health parameters in tundra caribou. An important limitation of this study remains to be the sample size, which should be improved to early detect changes in health indicators. However, with either sampling method alone, the interpretation and understanding of the herd’s health would be more limited. Convenience sampling methods entail potential biases that need to be considered when analyzing these data. Continued efforts to maintain, improve and standardize sampling schemes for health surveillance is imperative to achieve a better understanding of the various mechanisms and determinants causing the widespread tundra caribou declines. Following the proposed framework, and with further and better-balanced data, thresholds for health outcome metrics could defined^17^, and relationship between the different health determinants and outcomes assessed with multivariate analyses. Surveillance strategies that combine captures, community-based sampling, participatory approaches, and local and Indigenous knowledge can greatly contribute to advance our knowledge on caribou and wildlife health.

## Materials and methods

### Study area, population, and sampling methods

The Dolphin and Union (DU) caribou is a migratory tundra caribou herd endemic to the Canadian Arctic (Nunavut and Northwest Territories), and is considered a separate conservation and management unit because its unique genetics, morphology, and ecology^59^. This herd has been steadily declining since approximately the 1990s–2000s, with the last estimate of 3815 in 2020 (95% CI 2930–4966)^22^; it is currently considered endangered in Canada (COSEWIC). The steepest herd decline (78%) was documented between 2015 and 2018^21^ (Fig. 2). Detailed information is provided in Supplementary Appendix S1.

There are three Inuit communities, Kugluktuk, Cambridge Bay and Ulukhaktok, that harvest DU caribou at different locations along its seasonal migration. A collaborative health monitoring program for this herd was initiated in 2015 and has been facilitated through the Kutz Research Group, the Hunter and Trappers associations (HTO/HTCs) from Kugluktuk, Cambridge Bay, Ulukhaktok and the Governments of Nunavut and Northwest Territories. It includes two different sources of convenience sampling, caribou hunted for subsistence or recreational purposes (guided hunting) and sampling from captured animals for monitoring purposes by the Government of Nunavut. The involvement of Inuit communities in sample and data collection was coordinated through HTO/HTCs and started at different times depending on funding sources (Table S1). An example of the data form distributed among hunters for the community-based sampling is provided as supplementary information (Supplementary information – Data form).

Captures were performed by helicopter to deploy satellite collars on adult females^20^. In both community-based sampling and live-captured caribou, sampling methods were standardized following modified protocols of the CircumArctic Rangifer Monitoring and Assessment Network (CARMA)^60^. The specific sample list collected, methods of management and storage of samples and research permits are specified in Supplementary Appendix S1.

Health data from the DU herd from previous periods were available through the CARMA network (Don Russell). This included data of caribou body condition and health from government surveys between 1987 and 1991^61^, and data from a research study that took place between 2001 to 2003 on caribou harvested by local hunters in a similar way as hunters harvest animals for subsistence^62^.

All samples were analyzed under the certificate of Animal Use Protocol (C18-0093) that included the use of tissues and analyses from convenient samplings, approved by the Animal Care Committee from the University of Calgary. None of the tissues or cadavers used or analyzed in this study were derived from live animals purposely sourced or killed for the study.

### Sample analyses and health indices

**Figure 5:**
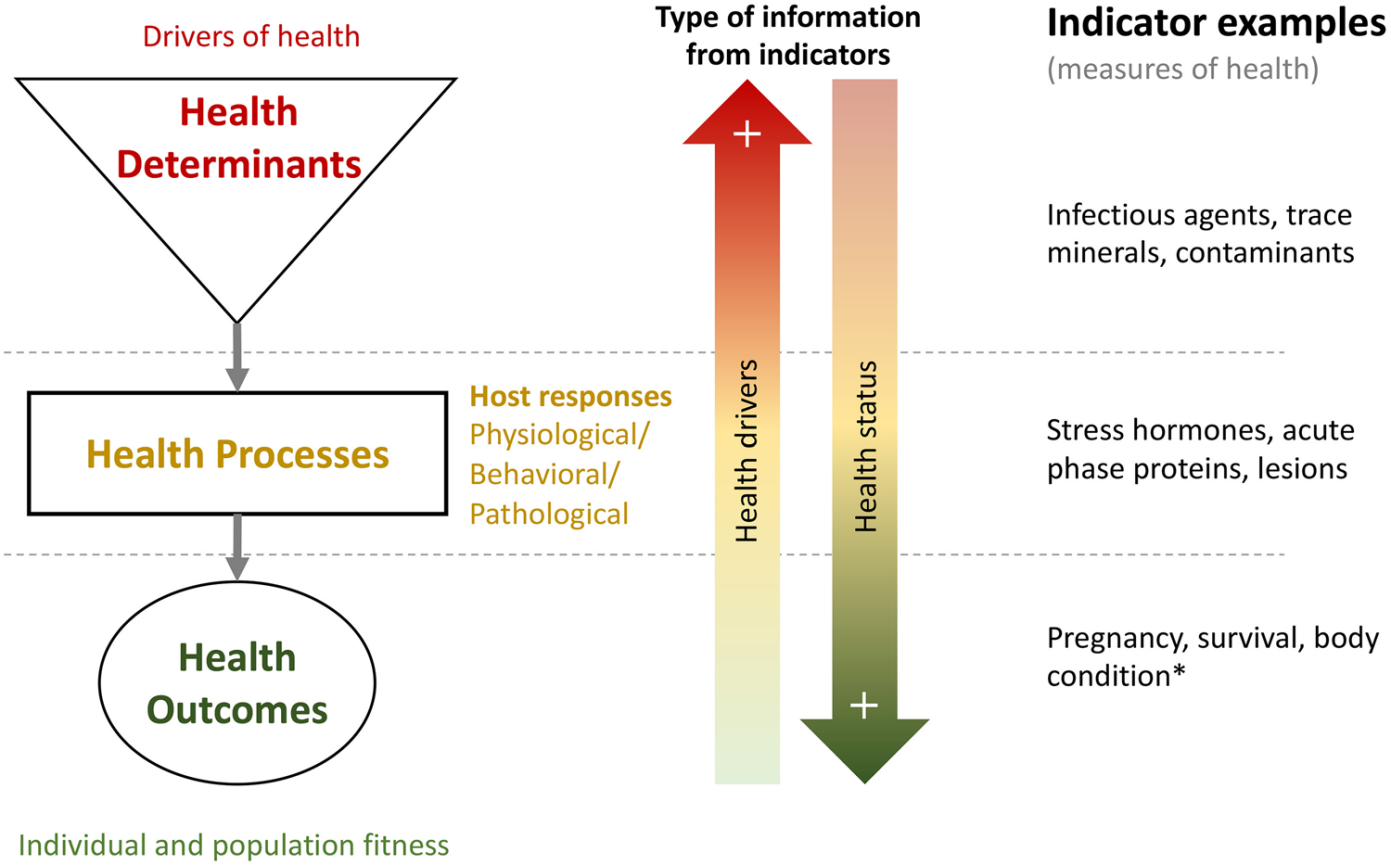
Health indicators framework. Classification and examples of health indicators as health determinants, health processes (host responses to determinants) and health outcomes showing a directional relationship in a gradient of information between health drivers and health status. This diagram depicts the main relationships between indicator class types but does not intend to encompass the whole theoretical complexity behind this framework. *Body condition may be considered as a health process as well, see full text for justifications.

We applied a health framework that classifies health indicators into health determinants, health processes and health outcomes (Fig. 5, Table 4). This framework elaborates upon previous concepts on health assessments and surveillance^18,19^. Briefly, health determinants can be considered as extrinsic or intrinsic factors that can promote or affect health^15,18^. Health processes are the animal’s physiological, pathological or behavioural processes that occur in response to determinants, termed ‘host responses’ here. These responses, e.g. stress hormones or acute phase proteins, are often not specific to a single determinant and therefore offer broader information about possible drivers of health. Finally, health outcomes are the result of the interaction of determinants of health and the resilience of animals, and include those parameters that are associated with fitness, such as reproduction or survival. For capital breeders, such as caribou, energy stores are critical and can be considered as a proxy for fecundity^63,64^, thus we used this indicator as a health outcome in this work.

Health outcomes provide the most relevant information needed for managers to anticipate the herd trajectory. Health outcomes alone, however, do not inform about the underlying drivers and processes leading to the health outcome. Parameters that measure health processes may provide some insights into the health status and may (or may not) inform on the determinants that triggered that response. Determinants of health, on the other hand, are those factors that are interacting to influence wildlife health and could be a possible target for management. In this study we first focus on analyzing health outcomes to assess the health status trends, and then use the information provided by metrics on responses and potential determinants to identify what drivers may be operating on the herd’s health.

### Health outcomes

Health outcomes included body condition, pregnancy status and data on survival (Table 4). Body condition metrics in harvested animals included the percentage of metatarsus marrow fat, kidney fat index (KFI), back-fat depth and a hunter qualitative assessment following the CARMA protocol^36^. In live-captured animals, a body condition score that range from 1 to 12 was derived from the palpation of different body parts (Supplementary Appendix S2).

Pregnancy status of caribou sampled in early spring (April and May) was inferred from fecal progesterone levels, following previously described protocols, and establishing within-population thresholds for non-pregnant females at < 200 ng/g and pregnant females at > 700 ng/g of wet feces^65^.

Where data collection methods were comparable, we compared our results on KFI and pregnancy rates in adult females in early spring with data from the DU caribou herd in 1987–1991 and 2001–2003.

### Health processes (host responses to determinants)

Physiological stress was measured indirectly using cortisol and its metabolites in hair and feces of the animals. Fecal stress hormone levels reflect physiological status from approximately the last 24–48 h before sample collection. Hair values largely reflect the accumulation of cortisol during the hair growth period from late spring to fall, representing conditions over several months during the previous summer and fall, and possibly also reflecting stressors during the non-hair growing season^31^. Cortisol concentrations (hair from the neck) and corticosteroid metabolites (feces) were quantified by enzyme immunoassay^32^ (Supplementary Appendix S3). Hair cortisol from 2015–2017 to 2018–2021 was analyzed in different laboratories (Supplementary Appendix S3). Part of the results on hair cortisol were previously published ^31^.

### Health determinants

For the assessment of pathogen exposure, blood collected with filter paper from both captured and hunted animals was eluted for antibody detection^66^. The presence of antibodies against selected pathogens, including α-herpesvirus, pestivirus, *Brucella*, *Erysipelothrix rhusiopathiae* and *Neospora caninum*, was tested assuming the 1:10 dilution of filter paper eluates^66^ (see Table S2 for test and laboratory information). The selection of pathogen assays was based on previous knowledge from this herd or other tundra caribou herds^24,39^. Part of the results on pathogen exposure were previously published (see footnote on Table 1) ^24^.

To estimate the intensity of infection of *Besnoitia tarandi*, we used the maximal density of bradyzoite-containing cysts (mdc) from the anterior aspect of the mid-third portion of the metatarsal skin region^40^ (Supplementary Appendix S4). We used the modified Baermann technique for the quantification of protostrongylid larvae from frozen feces^67^, but larvae of *Parelaphostrongylus andersoni* and/or *Varestrongylus eleguneniensis* were not differentiated^68^.

We determined the element concentrations in the hair from the neck collected from 2018 to 2021 using inductively coupled plasma mass spectrometry (ICP-MS) at the Alberta Centre for Toxicology. The methods used and the panel of trace minerals and contaminants analyzed are described in detail in Supplementary Appendix S5.

### Analyses for ancillary data

Caribou sampled were confirmed to belong to the Dolphin and Union herd by genetic analyses or, in the absence of these data, on sampling location and morphological traits described by harvesters^20^ (Supplementary Appendix S6). The age of caribou was assessed by analyzing the cementum annuli from an incisor or by the tooth eruption pattern of the incisors^36^. Age class was assigned as calf for < 12 months, yearling for 13–24 months, subadult for 25–36 months and adult for 37 months or greater.

### Data analyses and assessments

We performed statistical analyses to assess differences on health indices between the sampling source, sex of the animals, year of sampling or period of sampling in the case of historical data comparisons. First, we explored the distribution of data and its structure in relation to possible sources of covariation. Most samples were from adult caribou (88.2% of the samples, n = 247) and, therefore, we only used this age category for all our assessments. Because the dataset was highly unbalanced between years regarding the method and season of sampling or sex, and continuous variables were not normally distributed, we used univariate tests and subsets of data for statistical analyses. The specific sample sizes used for each analysis are showed in the results section together with the statistics of the tests.

For hair cortisol analyses, data derived from hair samples were corrected to the corresponding hair-growing year, thus samples collected from January through to spring were re-assigned to the previous calendar year when the hair would have been growing. We also excluded hair cortisol results corresponding to 2015 (hair samples collected in the fall 2015 and spring 2016) for statistical analyses because they were analyzed with a different test and in a different laboratory.

To evaluate differences between the two sampling sources, hunted caribou and captured caribou, we used a subset of data with adult DU caribou sampled in the same year and the same season (April and May 2018, n = 86). For this purpose, we used Fisher’s Exact Tests and Chi-square tests for independence to assess differences by sampling source in pregnancy status, exposure to pathogens and protostrongylid larvae detection; and Wilcoxon–Mann–Whitney tests to assess sampling source differences in fecal/hair cortisol, and hair trace minerals. Differences between sample sources were not assessed for *B. tarandi* cyst detection because we only had data from hunted caribou (data from an invasive skin sample), and for body condition because it was recorded differently for live (palpation) versus harvested (KFI, marrow fat, back-fat) animals.

To assess sex differences of health indices we used a subset of data including adult caribou from all years for health determinants, assuming no significant seasonal variability, and a subset of data for cortisol analyses that included adult caribou from early spring (April and May) 2018. Differences between years were assessed in the overall sample set of adult caribou in early spring (April and May), selecting females or a specific sampling source (hunted and captured) depending on the number of observations and only whenever these groups were not significant in previous assessments. We used Fisher’s Exact Tests and Chi-square tests for independence when variables were categorical and non-parametric Kruskal–Wallis and Wilcoxon–Mann–Whitney tests when variables were continuous.

Pregnancy rates and body condition of adult females in spring were compared among sampling periods (2015–2019, 2001–2003 and 1987–1992) using Chi-square and Kruskal–Wallis tests, respectively.

All analyses and data figures were done using the R software version 4.1.0^69^, and significance level were set at 0.05 in all tests.

### Supplementary Information

Supplementary Information.

### Supplementary Information

Supplementary Information.

## Data Availability

The datasets generated during and/or analysed during the current study are available from the corresponding author on reasonable request.
