# Supplementary material for: An integrative and multi-indicator approach for wildlife health applied to an endangered caribou herd
Source: Sci Rep. 2023 Oct 2;13:16524. doi: 10.1038/s41598-023-41689-y (PMC10545743; doi:10.1038/s41598-023-41689-y)
Supplement: Supplementary file 2 — Supplementary Information. [file 41598_2023_41689_MOESM2_ESM.pdf]

HOW TO COLLECT DU CARIBOU SAMPLES

1) BLOOD ON FILTER PAPER (as soon as the animal is killed)

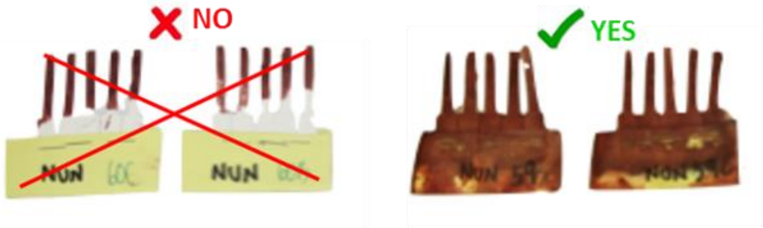

All strips soaked

Dry at the air

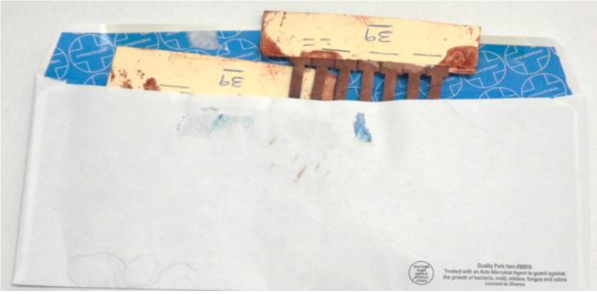

Close the envelope

BAG 1

2) SKIN WITH FUR

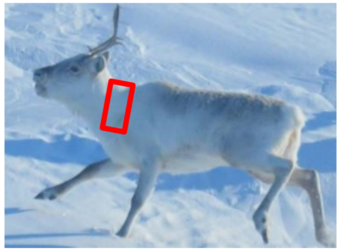

BAG 2

10 cm/4 inch

10 cm
4 inch

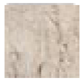

3) DROPPINGS From the butt

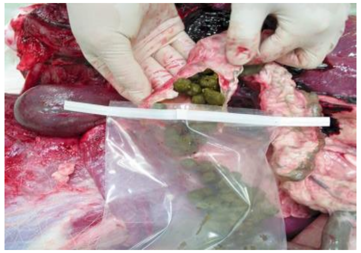

BAG 3

4) LEFT KIDNEY + FAT

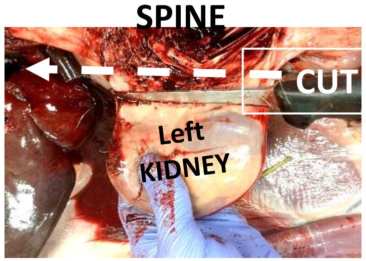

BAG 4

Cut at the poles of the kidney, perpendicularly to the spine (white line)

5) PIECE OF SPLEEN

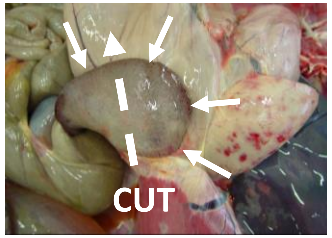

BAG 5

10 cm/4 inch

6) LEFT HIND LEG or METATARSAL BONE

TAG Fill-in the tag!

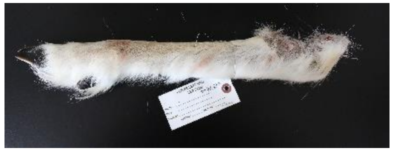

7) JAW or FRONT TEETH

TAG Fill-in the tag!

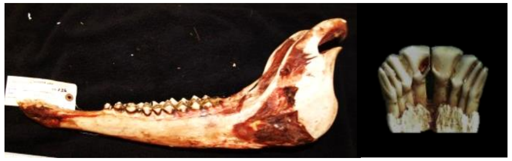

SAMPLE KIT BAG

+ ANYTHING STRANGE!

8) MEASURE BACK FAT

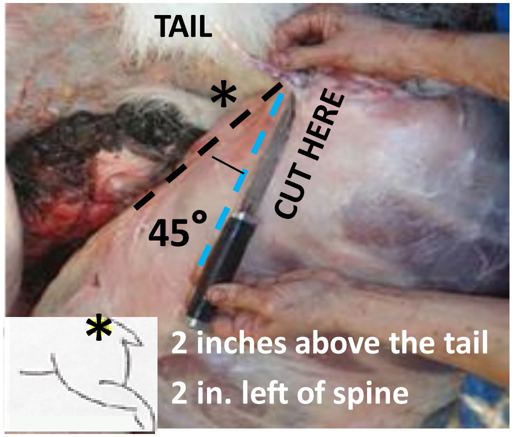

Measure BACK FAT using the ruler on the LEG TAG

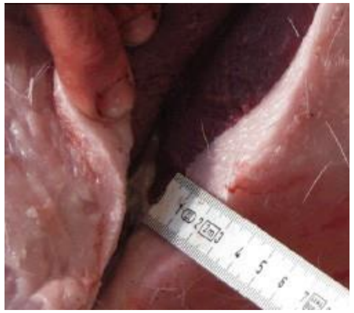

AT THE END

EVERYTHING

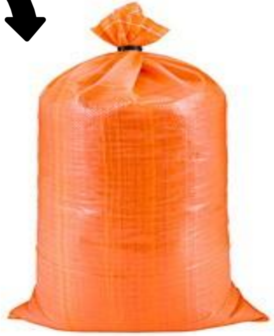

Fill-in the FORM on the back side. Return it in the pocket and close it !

DOLPHIN AND UNION CARIBOU SAMPLE COLLECTION

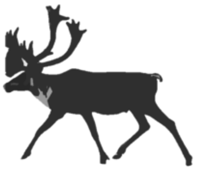

ANIMAL ID ☐

COMMUNITY : ☐

HUNTER'S NAME : ☐ GUIDE'S NAME: ☐

CONTACT: ☐

KILL DATE : ☐ / ☐ / ☐
dd mm yy

TYPE OF HUNT : Community ☐ Individual ☐ Sport ☐

KILL LOCATION : ☐
(for example: Nunatunek)

LATITUDE N ☐ LONGITUDE : W ☐

SEX : Female ☐ Male ☐ Unknown ☐

If female specify if: pregnant ☐ lactating ☐ none ☐ unknown ☐

AGE CLASS : Calf of this spring ☐ Yearling ☐ 2 y old or more ☐ Unknown ☐

BODY CONDITION : Really fat ☐ Fat ☐ Not bad ☐ Skinny ☐

BACK FAT : Put a mark on the scale

Inches

Centimeters

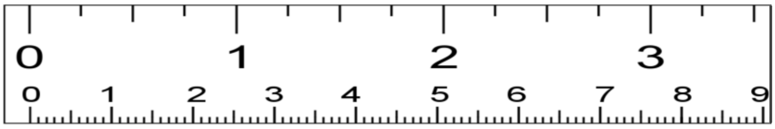

HERD SIZE N° : ☐ N° CALVES : ☐ N° YEARLINGS : ☐
all animals

SAMPLES COLLECTION (check the box when collected):

BLOOD ☐ SKIN+FUR ☐ DROPPINGS ☐ JAW ☐ SPLEEN ☐ LEFT KIDNEY ☐ LEFT HIND LEG ☐

ABNORMAL TISSUE specify: ______________________________

COMMENTS: ______________________________

____

____
